# Sub-100 nm manipulation of blue light over a large field of view using Si nanolens array

**DOI:** 10.1515/nanoph-2025-0413

**Published:** 2025-11-25

**Authors:** Zhiyuan Shi, Wei Jiang, Yanqing Lu, Weihua Zhang

**Affiliations:** College of Engineering and Applied Sciences, MOE Key Laboratory of Intelligent Optical Sensing and Manipulation, Nanjing University, Nanjing 210023, P.R. China; State Key Laboratory of Analytical Chemistry for Life Science, and Jiangsu Key Laboratory of Artificial Functional Materials, Nanjing University, Nanjing 210093, P.R. China

**Keywords:** super-resolution, nanofocusing, Si nanolens array, inverse problem

## Abstract

This study presents a super-resolution light manipulation technique in the near-field region of a silicon nanolens array in the blue spectral range using a computer-generated holography technique. It allows us to focus light into a spot below 70 nm at arbitrarily given positions within the entire lens array using modulated incident fields. To achieve this, an inverse design algorithm is developed using multiaxis high-order Gaussian beam expansion. It effectively corrects aberrations in off-axis focal spots within each nanolens unit, resulting in high-quality nanofocused beams with an extended depth of focus. By superimposing discrete nanofocused spots, we can further synthesize complex intensity patterns across multiple nanolens units, achieving an intensity profile resolution of 80 nm. This offers a promising approach for super-resolution photolithography using visible light.

## 1 Introduction

The manipulation of light beyond the diffraction limit is central to the field of nano-optics, offering profound implications for imaging, sensing, and photolithography. Over the past few decades, a variety of techniques have been developed, including superlenses [1], [2], [3], hyperlenses [4], [5], [6], and plasmonic structures [7], [8], [9]. These methods exploit the negative permittivity of noble metals to achieve nanoscale light confinement within the near-field regime. However, the potential for metal contamination of semiconductor devices makes these techniques unsuitable for many lithographic applications, particularly in the semiconductor industry. Concurrently, super-resolution techniques based on nonmetallic structures, such as immersion lenses [10], [11], [12], [13], [14], [15], [16] and photonic nanojet [17], [18], [19], have also been explored. While these approaches utilize transparent dielectric materials to reduce the wavelength, the achievable resolution is limited by the relatively low refractive index of these materials (typically below 2 in the blue and UV spectral ranges). To address these limitations, we introduced a new class of super-resolution lens made of a high-index, lossy material (e.g., silicon). This lens is capable of focusing light into a sub-50 nm spot at 405 nm [20], [21]. However, inherent material losses restrict both the size of the silicon lens and its corresponding field of view to extremely small dimensions (on the order of 100 nm).

To overcome this limitation, in this work, we extend the silicon nanolens to an array configuration, namely a silicon nanolens array (SNLA) and investigate the super-resolution computer-generated holography technique for such a device. Interestingly, similar concepts have been explored in plasmonics using array structures. For example, Zheludev and coworkers reported a subwavelength (180–200 nm) “hot spot” generation technique at arbitrary prescribed positions within a periodic meta-chessboard by modulating the incident beam’s phase [22]. Bergin Gjonaj et al. reported inverse algorithms for synthesizing focusing spots with a 420 nm linewidth at specified positions on a plasmonic nanohole array [23]. However, different to the hot-spot generation in plasmonic systems, which relies on the interference of 2D surface waves [24], the generation of super-resolution near-field holography by SNLA is a complex 3D scattering process. To simply this process, in this work, we borrow the idea from the work by Gu Group [25], which first uses an algorithm to construct points in space and then uses these point groups as basic units to form arbitrary super-resolution holographic patterns, which can used for nanophotolithography in the visible range.

## 2 Model

**Figure 1:**
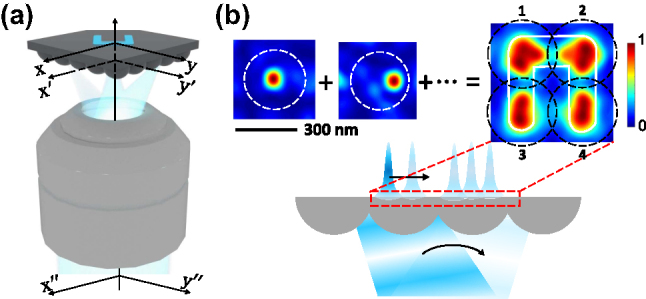
Principle of Si nanolens array. (a) Scheme of the optical system. (b) Super-resolution pattern generation with the Si nanolens array. The white solid line labels the boundary of the target function.

Figure 1 shows the principle of the SNLA-based super-resolution light manipulation technique. The SNLA consists of a 2D array of hemispherical Si nanolenses with a period of 270 nm and a radius of 150 nm. Modulated 450 nm light beams (e.g., amplitude or phase modulated) are first focused by an objective (N.A. = 1.45) on the entrance plane $\left(x^{\prime },y^{\prime }\right)$ , and the focused fields are further confined by the high-index SNLA structure to create target intensity patterns at super-resolution, achieving subdiffraction-limit focusing, in its output plane $\left(x,y\right)$ .

A 2D unit step function $I_{t}\left(x,y\right)$ is used as the target function:

(1) $$I_{t}\left(x,y\right)=\left\{\begin{array}{cc} 1, & \left(x,y\right)\in T,\\ 0, & \left(x,y\right)\notin T, \end{array}\right.$$

where, *T* denotes the exposure area, which can have any shape, any size at any given positions cross multiple lens unit in the SNLA. For example, in Figure 1, *T* has the shape of character “U” (initial of University) over 4 adjacent lens units.

Intensity superposition method is used to create the target optical patterns. More specifically, we generate the target intensity profile by adding a series of nanofocus spots, *I*_*k*,*ij*_ at positions $\left(x_{i},y_{j}\right)$ in lens units *k* (*k* = 1, 2, …, *N*) subsequently, as illustrated in Figure 1(b). In practice, this can be realized by modulating the beam in sequence. The question then becomes what are the right incident beams for given target function, $I_{t}\left(x,y\right)$ .

## 3 Inverse design of an arbitrary super-resolution pattern

**Figure 2:**
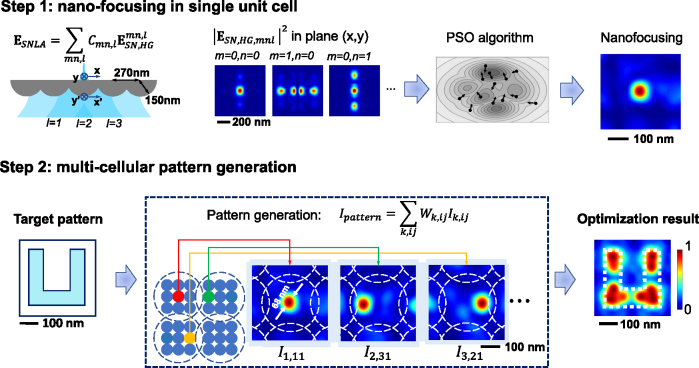
Diagram of the 2-step optimization algorithm for super-resolution pattern generation. In the first step, nanofocus spots are generated using HG beams, and in the second step, super-resolution patterns are synthesized using the nanofocus spots in the first step.

To solve this inverse problem, we developed a two-step algorithm, as shown in Figure 2. The first step uses an optimization algorithm based on high-order Gaussian beam expansion to generate a series of nanofocus spots at different locations. The second step uses these nanofocus spots as “basis functions” to synthesize the target intensity distribution.

### 3.1 The first step: nanofocus generation

The goal here is to find incident $E_{\text{in}}^{\left(k\right)}$ , which can generate the smallest focus spot $E_{\text{SNLA}}^{\left(k\right)}$ at a given position **r** at the output plane of lens unit *k*. Since this is a linear system, we have

(2) $$\begin{array}{ll} E_{\text{SNLA}}^{\left(k\right)}\left(r\right) & =\int \text{d}r^{\prime }P_{\text{SNLA}}\left(r,r^{\prime }\right)\\ & \;\times \int \text{d}r^{\prime \prime }P_{\text{obj}}\left(r^{\prime },r^{\prime \prime }\right)E_{\text{in}}^{\left(k\right)}\left(r^{\prime \prime }\right), \end{array}$$

where, **P**_obj_ and **P**_SNLA_ are, respectively, the transfer functions of the objective and the SNLA, **r**, **r**′, and **r**″, respectively, denote positions in the incident field plane $\left(x^{\prime \prime },y^{\prime \prime }\right)$ , the focal plane $\left(x^{\prime },y^{\prime }\right)$ of the objective, and the output plane $\left(x,y\right)$ of the SNLA.

To solve this problem, we discretize the $\left(x,y\right)$ , $\left(x^{\prime },y^{\prime }\right)$ , and $\left(x^{\prime \prime },y^{\prime \prime }\right)$ plane into *N*_1_, *N*_2_, and *N*_3_ square meshes, respectively. Then, $E_{\text{in}}^{\left(k\right)}$ and $E_{\text{SNLA}}^{\left(k\right)}$ become matrices, and Eq. (2) can be rewritten as

(3) $$E_{\text{SNLA}}^{\left(k\right)}=M_{\text{SNLA}}M_{\text{obj}}E_{\text{in}}^{\left(k\right)}.$$

Here, **M**_obj_ and **M**_SNLA_ are the matrix-form of transfer function **P**_obj_ and **P**_SNLA_, which are 3*N*_2_ × 3*N*_3_ and 3*N*_1_ × 3*N*_2_ in size, respectively.

To reduce the size of parameter space of Eq. (3), we rewrite **E**_in_ as the supposition of Hermite–Gaussian (HG) beams with different axial positions,

(4) $$E_{\text{in}}^{\left(k\right)}=\underset{mn,l}{\sum }C_{mn,l}^{\left(k\right)}E_{\text{HG},mn,l}.$$

Here, **E**_HG,*mn*,*l*_ is the complex field distribution of the *mn*th order HG beam centered at $\left(x_{l}^{\prime \prime },y_{l}^{\prime \prime }\right)$ , which corresponds to the axis of the *l*th lens unit at $\left(x_{l},y_{l}\right)$ . In this work, *l* = *k*, *k* ± 1. In other words, the axes of the HG beams are at the centers of *k*th lens unit and its neighbor units. Insert Eq. (4) into (3),

(5) $$E_{\text{SNLA}}^{\left(k\right)}=\underset{mn,l}{\sum }C_{mn,l}^{\left(k\right)}E_{\text{HG},mn,l}^{\prime \prime }.$$

Here, $E_{\text{HG},mn,l}^{\prime \prime }$ is focused HG beam **E**_HG,*mn*,*l*_ by the system (i.e., the objective and the SNLA subsequently),

(6) $$E_{\text{HG},mn,l}^{\prime \prime }=M_{\text{PNSTF}}M_{\text{obj}}E_{\text{HG},mn,l}.$$

To calculate $E_{\text{HG},mn,l}^{\prime \prime }$ , we first calculated the focused field $E_{\text{HG},mn,l}^{\prime }=M_{\text{obj}}E_{\text{HG},mn,l}$ by the objective at $\left(x^{\prime },y^{\prime }\right)$ plane using Wolf’s integral method [26]. Then, $E_{\text{HG},mn,l}^{\prime }$ are obtained using the finite-difference time-domain method (FDTD) with $E_{\text{HG},mn,l}^{\prime }$ as the incident. Here, the refractive index of Si is adapted from Palik’s results (*n* = 4.678 + *i*0.148).

After knowing $E_{\text{HG},mn,l}^{\prime \prime }$ , the question becomes how to figure out values for $C_{mn,l}^{\left(k\right)}$ , which lead to the smallest focus spot at a given position **r** in the lens unit *k*. This can be solved with standard optimization algorithm. In this work, the particle swarm optimization (PSO) algorithm is used to search the minimum value of the full-width at half-maximum (FWHM) of the intensity profile of the focused field ${\left|E_{\text{SNLA}}^{\left(k\right)}\right|}^{2}$ . PSO is a global optimization algorithm inspired by the movement behavior of bird flocks and fish schools. It models a population of particles, where each particle represents a candidate solution in the parameter search space. Today, this method is mature, with numerous software packages and open-source code available, and it is widely used in various optical calculations [27]. Using this method, we can generate nanofocus spots at precisely defined positions $\left(x_{i},y_{i}\right)$ in lens unit *k*, labeled as $E_{\text{SNLA}}^{\left(k\right)}\left(x_{i},y_{i}\right)$ . For convenience, we use *E*_*k*,*ij*_ to denote $E_{\text{SNLA}}^{\left(k\right)}\left(x_{i},y_{i}\right)$ in the following part.

### 3.2 The second step: nanopattern synthesis

In this part, we use $I_{k,ij}={\left|E_{k,ij}\right|}^{2}$ as the basis function to synthesize complex super-resolution intensity patterns *I*_*s*_ via linear superposition

(7) $$I_{s}=\underset{k,ij}{\sum }W_{k,ij}I_{k,ij}.$$

Here, *W*_*k*,*ij*_ are the weight for each *I*_*k*,*ij*_. Similar to the first step, PSO algorithm is used to search the best weight for generating target distribution *I*_*t*_.

It is important to note that the absolute intensity difference $\left|I_{t}-I_{s}\right|$ is not directly suitable as a merit function for optimization. This is because *I*_*t*_ is zero outside the target area, while *I*_*k,ij*_ exhibits a nonzero background. To address this issue, we employ the intensity contrast, rather than the absolute intensity difference, as the basis for our merit function.

(8) $$D=\max\left(I_{s}\right)-\left(\frac{\underset{\left(x_{i},y_{i}\right)\in T}{\sum }I_{s}\left(x_{i},y_{i}\right)}{N_{\text{in}}}-\frac{\underset{\left(x_{i},y_{i}\right)\notin T}{\sum }I_{s}\left(x_{i},y_{i}\right)}{N_{\text{out}}}\right).$$

Here, *N*_in_ and *N*_out_ are the number of meshes inside and outside the target area *T* defined in Eq. (1), respectively. In addition, intensity variation inside the target area needs to be small, and therefore, we define the variation function

(9) $$V=\frac{\underset{\left(x_{i},y_{i}\right)\in T}{\sum }\left(\max\left(I_{s}\right)-I_{s}\left(x_{i},y_{i}\right)\right)}{N_{\text{in}}}.$$

The merit function can be defined as the superposition of D and V

(10) $$f_{m}=\alpha D+V.$$

Here, *α* is the weight of the intensity variation, and it is normally set to 5 in this specific case. In the optimization, we minimize the merit function by searching *W*_*k*,*ij*_ in the whole parameter space using PSO.

## 4 Results

### 4.1 Nanofocus spots

**Figure 3:**
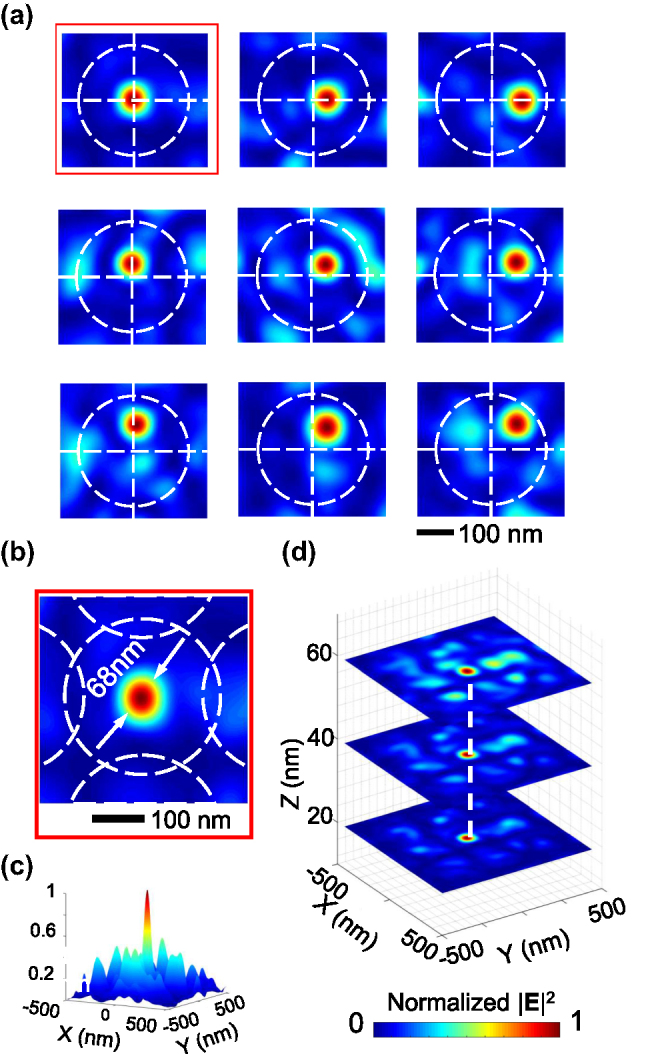
Nanofocusing generation within a single nanolens unit. (a) Nanofocus spots created at 9 different positions of the top-right quadrant of a lens unit. (b) The zoomed-in view of spot at (0 nm, 0 nm) in (a). (c) 3D view of the intensity map. (d) Field distribution of the spot at (0 nm, 0 nm) at different heights (*z* = 20, 40, and 60 nm).

Using the optimization method, we are able to focus light into a nanoscale spot at a given position in an arbitrarily pointed lens unit *k*. As shown in Figure 3(b), at the center point of the lens unit, the spot size (i.e., the full width of half maximum (FWHM) of the ${\left|E_{k,ij}\right|}^{2}$ ) reaches 68 nm (less than *λ*/6). In addition, this nanofocus enjoys a large depth of focus (DOF). It extends for several tens of nanometers away from the surface of the SNLA structure without losing much of its intensity (Figure 3(d)), making it useful for many applications, particularly in photolithography.

Such a super-resolution focus spot can be obtained at any pregiven locations. We run the nanospot synthesis algorithm across the whole lens unit with a step of 5 nm, and focus spots around 70 nm were successfully obtained at each of these positions within the area $\sqrt{{\left|x_{i}\right|}^{2}+{\left|y_{i}\right|}^{2}}\leq 105\;\text{nm}$ . Figure 3(a) shows some typical examples at the upper-left quadrant of a lens unit. One can see that the focus size stays constant, and the backgrounds are kept low (less than half of the focus spot). Same results can be produced in the rest three quadrants.

### 4.2 Synthesis of complex nanopatterns

**Figure 4:**
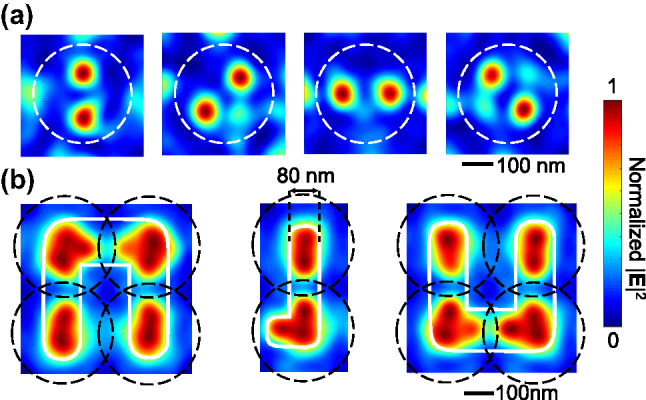
Complex pattern synthesis using nanofocusing spots. (a) Synthesized nanospot pairs within a lens unit. (b) Synthesized “N,” “J,” “U” patterns across multiple lens units. The line resolution reaches 80 nm.

With the help of the nanofocus spots, complex intensity distributions can be synthesized. Here, we first test the pattern generation capability using rotating nanospot pairs within a single lens unit, as shown in Figure 4(a). Well-separated spots can be squeezed in just one lens unit, the spot size stays at 70 nm, and the background stays low (i.e., below half of the peak intensity).

We further test the capability of SNLA with more complex and larger pattern across multiple lens units. Figure 4(b) shows an example, in which “N,” “J,” and “U,” the initials of Nanjing University, are synthesize. Here, the line width is approximately 80 nm, slightly larger than the size of nanofocus spots. There are breaks at the junctions between lens units because no nanofocus spots can be formed at the very edge of a lens unit (namely, area $\sqrt{{\left|x_{i}\right|}^{2}+{\left|y_{i}\right|}^{2}}>105\;\text{nm}$ ).

Compared to previously reported plasmonic holography results, the SNLA method proposed here shows clear advantages. For plasmonic nanohole arrays, the spot size is 200 nm at a wavelength of 852 nm. For the continuous metal substrates, the spot size is even larger, reaching 400 nm at a wavelength of 633 nm. The resolution of these methods is theoretically limited by the effective wavelength of surface plasmon polaritons (SPPs), which is extremely difficult to achieve sub-100 nm resolution. This makes them unsuitable for nanolithography-related applications.

## 5 Discussion

To achieve the above results, the model is fine-tuned to balance the main parameters, including resolution, field of view, and background level. Different choices of the basis functions, merit function, and geometrical parameters of the SNLA have been tested. The details are in the following.

### 5.1 Multiaxial HG beam expansion technique significantly improves the quality of off-axis focusing spots

As aforementioned, in the first optimization step, multiple set of HG beams with different axes are used to generate the incident light instead of single set of HG beams at one axis. Although theoretically HG beams form a complete set of basis functions, the introduction of multiaxial expansion significantly improved the quality of the synthesized beam, especially for the spots not at the center position of a lens unit.

**Figure 5:**
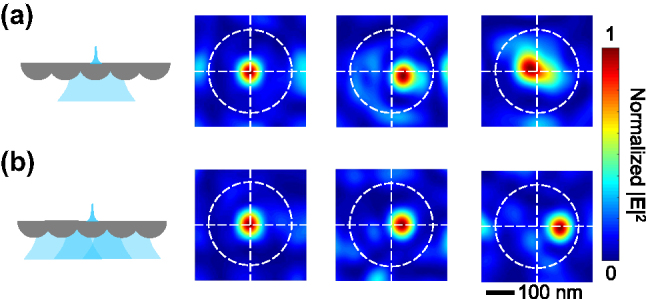
Comparison between multiaxial and single-axial HG beam expansion. (a) and (b) show the optimization result of nanofocus spot at *x* = 0, 45, and 90 nm with single-axial and multiaxial HG beam expansion technique, respectively.

Figure 5 illustrates the performance differences between multiaxial and single-axial Hermite–Gaussian (HG) beam expansion techniques. For direct comparison, focal spots are generated at three distinct target positions: (0 nm, 0 nm), (45 nm, 0 nm), and (90 nm, 0 nm). At the central position (0 nm, 0 nm), both methods produce a symmetric, sub-70 nm spot with a clean background. However, at (45 nm, 0 nm), the single-axial method yields an asymmetric spot, and at (90 nm, 0 nm), a focal spot cannot be formed. Conversely, the multiaxial HG beam expansion technique consistently generate symmetric, sub-70 nm focal spots with a clean background at all three positions. Furthermore, with the multiaxial HG beams, a nanofocus spot can be synthesized out to *x* = ±105 nm, closely approaching the lens unit edge. Consequently, only less than one-quarter of each SNLA unit’s area remains uncovered.

Nevertheless, “blind areas” still exist at the edge of the lenses. This leads to the break points when synthesizing large patterns across multiple lens units.

### 5.2 Balancing background and resolution

In addition to the nanofocus, the use of high-order HG beams also introduces background in the rest areas. To avoid undesired high background, a threshold value for the background is added in the first step of the optimization algorithm. Results show that a higher threshold value for the background will lead to smaller focus spot, and vice versa. In this work, we set the threshold at half of the intensity of focus point (i.e., the background is lower than half of the peak intensity of the focus point over the whole area).

### 5.3 Influence of the lens size

The performance of the SNLA is also related to its geometrical parameters, and the optimization result slightly varies when the diameter of lens unit size and the pitch size of the SNLA are changed. More specifically, we tested the cases with the radius of unit lens from 145 nm to 200 nm and the pitch size from 240 nm to 300 nm, and the size of focused spot varies in the range from 68 nm to 92 nm.

### 5.4 Influence of wavelength

Finally, it is worth noting that the spot size of nanofocus related to the wavelength of the light source. We also ran the optimization algorithm at 405 nm, but surprisingly, the size of the focus is 88 nm, considerably larger than the case of 450 nm. One possible reason is that the material losses are considerably larger than the case of 405 nm, and as a result, the effects of the high order HG modes are considerably weaker.

### 5.5 Practical considerations

To realize the aforementioned theoretical scheme, experimental implementation remains highly challenging, as it requires the fabrication of submicrometer-scale hemispherical arrays. Fortunately, with the rapid progress of nanotechnology in recent years, experimental solutions have emerged. Using thermal probe lithography, three-dimensional relief structures can be fabricated in photoresist materials with a precision of sub-10 nm [28], and these relief patterns can subsequently be transferred onto silicon substrates through etching processes. Researchers have achieved dense lines in silicon with a 14 nm half-pitch and an edge roughness of 2.6 nm. [29].

It should be noted that, unlike resonant designs whose optical performance is extremely sensitive to fabrication accuracy, SNLA is nonresonant and not sensitive to nanoscale variations of the geometrical parameters. To verify this, we intentionally introduced various random variations into the SNLA model, including the radii of the nanolenses along the *x* and *y* axes and the positions of their axes. The results show that the performance is insensitive to 10 nm variations; the changes in focal spot size, depth of focus, and sidelobe levels are negligible. Since a dimensional deviation of 10 nm corresponds to an optical path difference of approximately 40 nm, below one-tenth of the wavelength, this is not surprising. Considering that the precision of thermal probe fabrication techniques can reach the sub-10 nm level, the SNLA’s high tolerance for fabrication accuracy makes it experimentally feasible.

In summary, we developed a near-field computer generated holography technique for silicon nanolens array (SNLA), a nanostructured thin film of a lossy, high index material at 450 nm, with the goal of generating arbitrary super-resolution optical patterns. To accomplish this, we developed an inverse algorithm, employing multiaxial Hermite–Gaussian (HG) beam expansion, to calculate the input fields required for prescribed target patterns. This method enabled the generation of sub-70 nm (less than *λ*/6) focal spots at arbitrary positions, with a depth of focus exceeding 60 nm. Moreover, the complex super-resolution optical patterns with a linewidth of 80 nm are synthesized by superposing these nanofocus spots. The achieved resolution is comparable to that of deep-ultraviolet (DUV) immersion lithography. Considering that 450 nm is in the blue light region, there are many mature photoresist systems and semiconductor light sources are available. This allows it to be naturally applied to the fabrication of various nanostructures, providing a novel, nonscanning pathway for near-field lithography.
